# Electron work function – a probe for interfacial diagnosis

**DOI:** 10.1038/s41598-017-08841-x

**Published:** 2017-08-29

**Authors:** D. Y. Li, Liqiu Guo, Lei Li, Hao Lu

**Affiliations:** Department of Chemical and Materials Engineering, University of Alberta, Edmonton, AB T6G 1H9 Canada

## Abstract

A poor interface or defected interfacial segment may trigger interfacial cracking, loss of physical and mechanical functions, and eventual failure of entire material system. Here we show a novel method to diagnose local interphase boundary based on interfacial electron work function (EWF) and its gradient across the interface, which can be analyzed using a nano-Kelvin probe with atomic force microscope. It is demonstrated that a strong interface has its electron work function gradually changed across the interface, while a weaker one shows a steeper change in EWF across the interface. Both experimental and theoretical analyses show that the interfacial work function gradient is a measure of the interaction between two sides of the interface. The effectiveness of this method is demonstrated by analyzing sample metal-metal and metal-ceramic interfaces.

## Introduction

For functional and structural multiphase materials, composites and thin films, the interface between adjacent substances or phases plays a crucial role in affecting performance of the material systems. It is challenging to directly evaluate interfacial bonding strength. Although transmission electron microscopy is widely used to characterize interfacial structure and defects^1, 2^, it is difficult to simultaneously identify detrimental interfaces or interfacial segments as risk carriers, which may trigger global failure of thin films, multiphase and composite materials. Computational simulation provides valuable information on interfacial behavior^3, 4^. However, such computational analysis is feasible only for limited situations and insufficient for guiding material design. Although there are various methods for interfacial bonding evaluation, e.g., tension tests, peeling tests, local pushing tests, scratching tests, and quantification of interfacial fracture energy^5–13^ etc., these methods only provide information on average or overall interfacial bonding for films or coatings and composites. Thus, they are unable to capture local detrimental interfacial segments which could trigger global failure. Micro-indentation tests are often used to evaluate local interfacial failure from formed kinks on load-displacement curves or local cracking at interfaces^13–16^. However, cracking of hard brittle films or coatings themselves may also result in kinks on the indentation curves, and interfacial cracking could initiate from the hard/brittle coatings rather than at interface. Thus, alternative methods that can provide information on local interfacial failure are highly desired.

In principle, the interfacial bonding is largely determined by the electron behavior that determines atomic interactions at interface^4, 17–19^. The mechanical behavior of metals is correlated to their electron work function (EWF or *φ*)^20–23^, which is the minimum energy required to move electrons at Fermi level from inside a solid to its surface^24^. A higher EWF corresponds to a more stable electronic state, which is related to the stability of mechanical and electrochemical states of a solid or its resistance to mechanical and electrochemical attacks. A material with a higher EWF has stronger atomic bonds, higher elastic modulus and electrochemical resistance^20–25^. EWF can also be an indicator of interfacial strength. An early study shows that lower interfacial EWFs corresponds to weaker interfaces^26^. Compared to grains, grain boundaries have lower EWF^27^, leading to faster degradation when exposed to corrosive environments and lowered resistance to mechanical attacks such as creep^28^. However, interfaces do not always show low EWF. Electron localization may enhance the interfacial bonding for coherent interfaces^29^. In our studies, it is noticed that the gradient of EWF across an interface appears to reflect the interfacial bonding strength. An interfacial segment with a lower EWF gradient, *dφ*/*dx*, is stronger than one with a high gradient. This phenomenon is observed for both metal-metal and metal-ceramic interfaces. Thus, a further look into the correlation between EWF and interfacial strength could lead to new methodologies for interfacial evaluation.

In this article, we show a novel approach to diagnose local interphase boundary based on interfacial electron work function (EWF) and its gradient across the interface, which can be analyzed using a nano-Kelvin probe with atomic force microscope. The effectiveness of this method is demonstrated by analyzing sample metal-metal and metal-ceramic interfaces. First-principle analysis is also carried out to look into interfacial electronic configurations. Efforts are made to elucidate mechanisms responsible for the variation in EWF across the interface and its correlation with the interfacial bonding.

Samples materials for this study include conventional 2507 duplex stainless steel (DSS), Fe-45Cr-5C cast iron and TiC/Co composite. Specimens were wet ground with SiC paper up to 2000 grit, and then mechanically polished using a 1.5 µm-diamond paste. The steel sample was electrochemically polished using a solution of HNO_3_:H_2_O = 1:1 for 20 sec under an applied voltage of 1.2 V. The electrochemical polishing helped distinguish ferrite and austenite phases. All specimens were ultrasonically cleaned in ethanol and dried by a N_2_ gas flow. Experiments were performed using Bruker MultiMode AFM 8 with PeakForce KPFM capability. Bruker magnetic material coated probes (MESP) with 2.8 N/m force constant were used for the magnetic force microscopy (MFM) image and work function measurements, while Bruker ScanAsyst-Air probes with 0.4 N/m force constant and diamond probes with 350 N/m force constant were used to measure the modulus and deformation, respectively.

First-principles calculation was conducted using the Vienna Ab initio Simulation Package (VASP) with projector-augmented wave (PAW) potential^30–34^. The generalized gradient approximation (GGA) with the exchange-correlation functional of Perdew-Burke-Ernzerhof (PBE) was employed^35^. An cutoff energy of 400 eV and dense k-points sampling with a Methfessel-Paxton smearing of 0.2 eV were used to guarantee high numerical accuracy for both energy and stress optimization. The global break condition for the electronic self-consistency was chosen as 1.0 × 10^–5^ eV per supercell for all calculations. Due to the ferromagnetic nature of Fe, the spin-polarized set is necessary for our calculations. To investigate austenite/ferrite interfaces, we constructed 1 × 2 × 3 supercells of Fe-FCC (*γ*–*Fe*: a = 3.442 Å, b = 6.884 Å, c = 10.326 Å) on its primitive cell (a = b = c = 3.442 Å), and 1 × 2 × 4 supercells of Fe-BCC (α − *Fe*: a = 2.8664 Å, b = 5.7328 Å, c = 11.4656 Å) on its primitive cell (a = b = c = 2.8664 Å). (001) facet of the Fe-FCC supercell was connected to (001) and (021) facets of the Fe-BCC supercells to build (001)γ||(001)α and (001)γ||(021)α interfaces, respectively. For the calculation of work function, the vacuum layers of 15 Å were inserted in these interfacial models. It should be mentioned that thickness of the *γ*−*Fe* cell and that of α − *Fe* cell are 10.326 Å and 11.4656 Å, respectively. Thus, total thickness of the interface model with the additional vacuum layer of 15 Å is equal to about 36.7916 Å. Such a size should be large enough for calculating the system’s properties under study. For sufficient convergence, the tolerance value in the geometry optimization is 1.0 × 10^−5^ eV/atom, which is small enough to ensure accuracy of conducted calculations.

**Figure 1:**
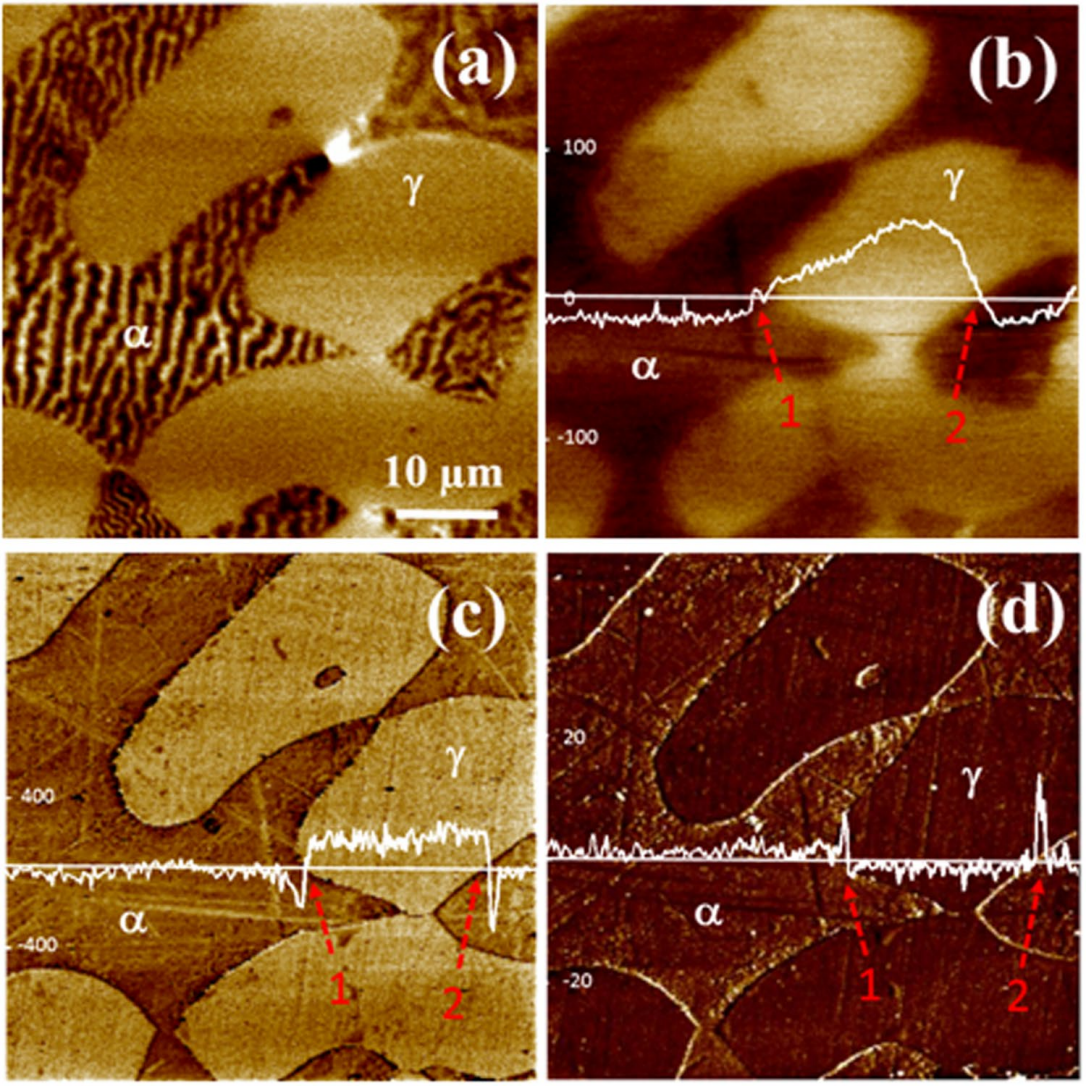
(**a**) A MFM image of the duplex stainless steel; (**b**) a work function map with a line profile; (**c**) corresponding modulus map with a line profile; and (**d**) a deformation map with a line profile.

Ferrite/austenite interface in a duplex stainless steel (DSS) was investigated as an example of metal-metal interfaces. Figure 1(a) presents a MFM image (Magnetic Force Microscopy) of the steel, confirming that it consists of ferromagnetic ferrite (α) and paramagnetic austenite (γ). Corresponding maps of work function, elastic modulus and deformation are given in Fig. 1(b–d). Line profiles of the properties are also provided. As shown, the austenite has a higher work function than the ferrite, accompanied with larger modulus and smaller deformation magnitude than those of the ferrite. Average values of the properties are given in Table 1. As shown in Fig. 1(b), the EWF line profile cresses two F/A interface locations, which changes gradually at location 1 while rapidly at location 2. At interface, the modulus decreases along with a larger deformation magnitude. The modulus is dependent on the atomic bond strength and lattice configuration. A deeper interatomic potential well corresponds to higher bond stability, rendering the atomic bonding stronger. This is also applied to interfacial zone with disordered structure but the interatomic potential is the same. In general, the higher the interfacial modulus, the stronger is the interface^36, 37^. As demonstrated, the interface with smaller *dφ*/*dx* at location 1 is stronger than the one at location 2. We examined more *α/γ* interfacial locations and present results in Table 2. As shown, the larger the work function gradient, the weaker the interface.

**Table 1:** Average values of work function, modulus and deformation of ferrite (α) and austenite (γ).

	Work function (eV)	Modulus (Gpa)	Deformation (nm)
Ferrite	4.95	163.8	2.2
Austenite	5.05	182.2	1.2

**Table 2:** *dφ*/*dx* and corresponding moduli and deformation magnitudes of six examined ferrite/austenite interfaces.

Location	*dφ*/*dx* (meV/µm)	Interfacial Modulus (GPa)	Interfacial Deformation (nm)
# 1	9.50	160.8	2.4
# 2	13.3	158.3	2.5
# 3	15.4	155.6	2.7
# 4	28.5	147.4	3.2
# 5	30.5	140.5	3.4
# 6	37.5	135.4	3.6

**Figure 2:**
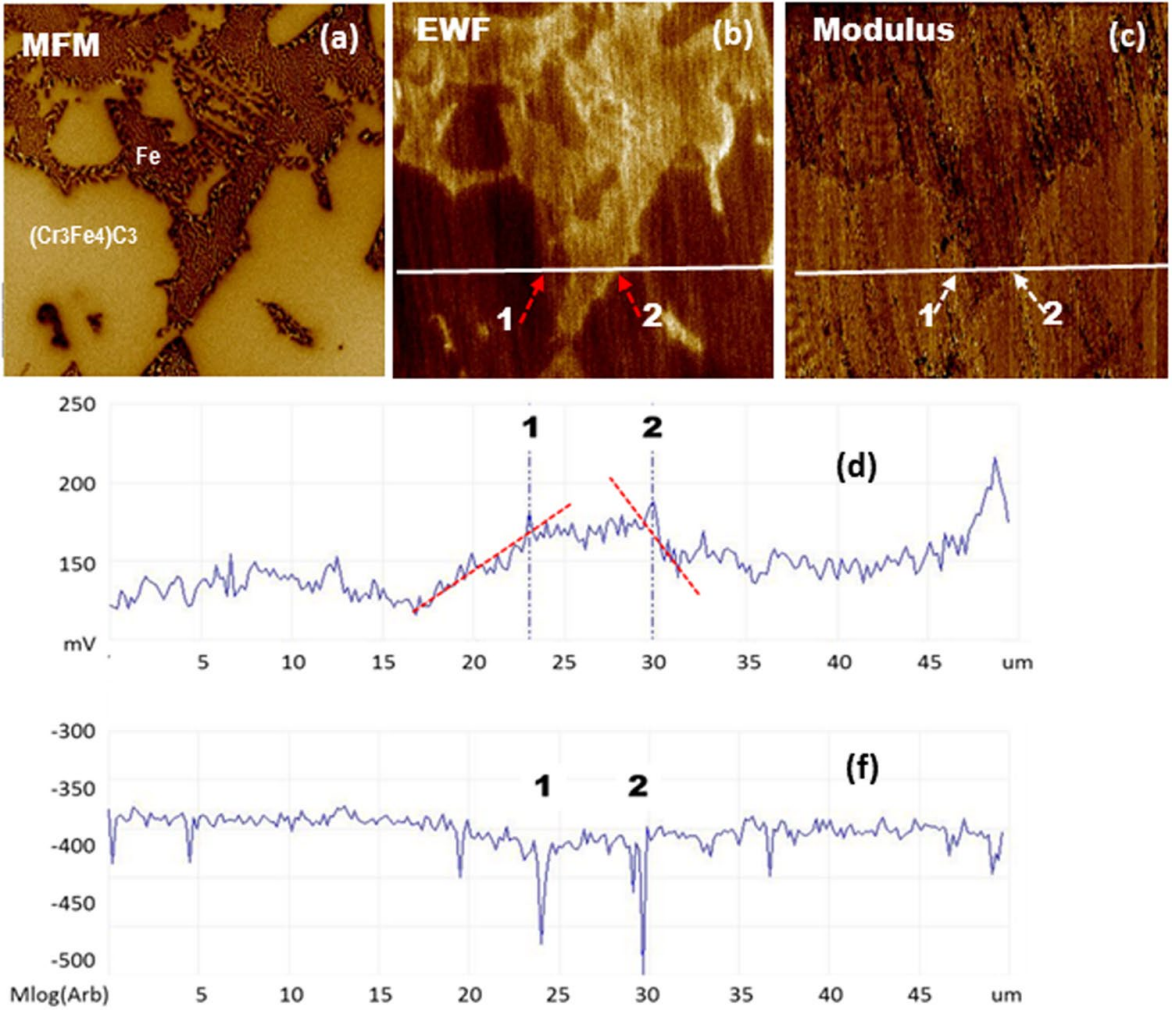
(**a**) a MFM image of a high-Cr cast iron, (**b**) corresponding work function map, (**c**) modulus map, (**d**) a line profile of work function, (**f**) a line profile of modulus.

**Figure 3:**
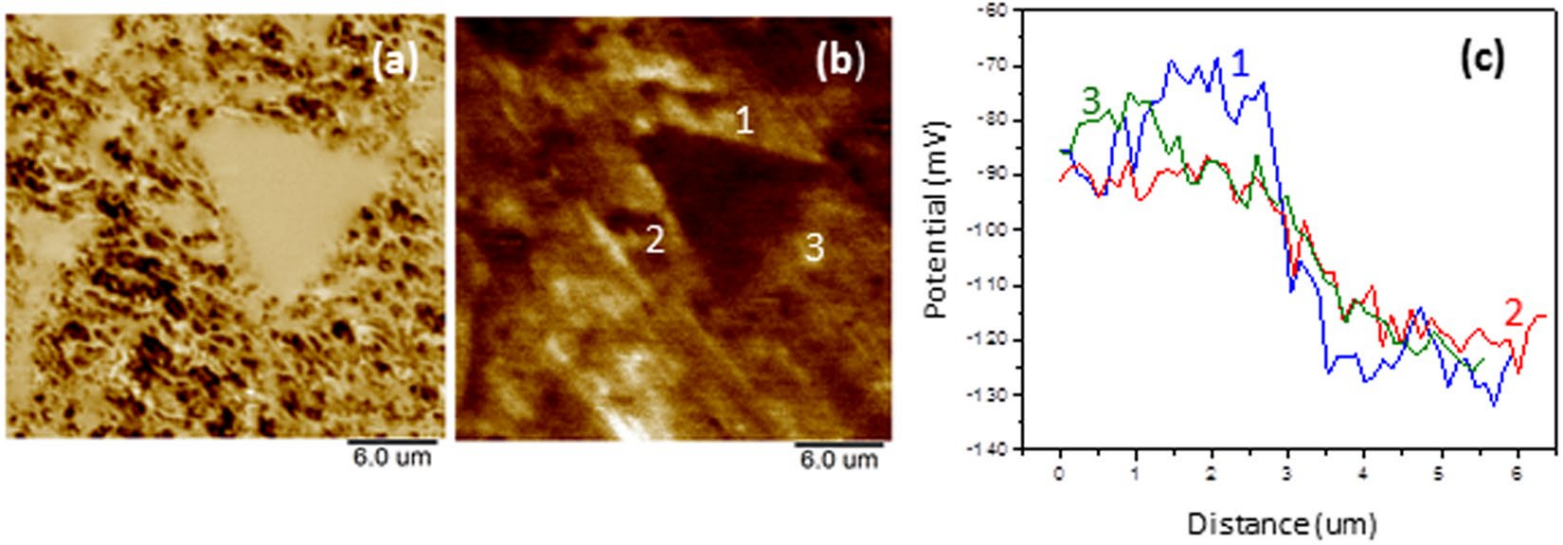
(**a**) a MFM map (magnetic) and (**b**) corresponding EWF map of a TiC in a Co matrix; (**c**) linear profiles of EWF across three TiC/Co interfaces; EWF slopes and corresponding modulus values are given in Table 3.

Metal-ceramic interfaces show similar phenomenon. Figure 2 illustrates interfaces between (*Fe*_3_ *Cr*_4_)*C*_3_ and ferrous matrix in a high-Cr cast iron. As illustrated, a larger *dφ*/*dx* results in a larger drop in modulus at location 2 (see Fig. 2(d and f)), corresponding to a lower mechanical stability of this interfacial zone. Similar situation is also observed in other metal-ceramic systems, e.g., TiC/Co interfaces as shown in Fig. 3, for which corresponding EWF gradients and interfacial moduli are given in Table 3.

**Table 3:** EWF slopes and corresponding modulus and deformation magnitude of three examined TiC/Co interfaces (shown in Fig. 3).

Interface location	EWF slope (meV/μm)	Modulus (GPa)
1	43.6	180
2	24.4	220
3	22.5	259

**Figure 4:**
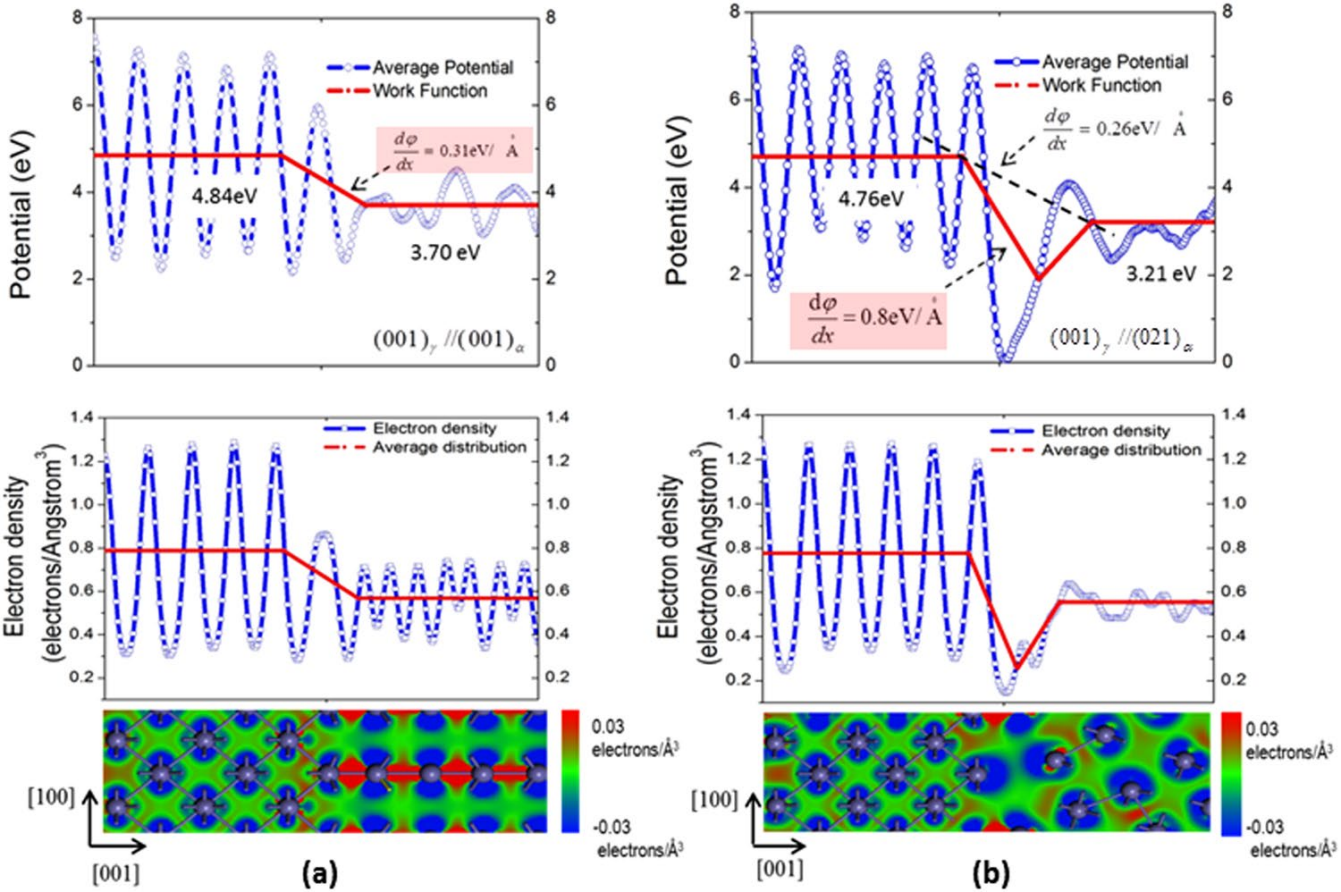
Electrostatic potential, EWF and electron configuration in the interfacial regions of (**a**) (001)_*γ*_//(001)_*α*_ interface with its binding energy = 7.758 eV/atom, and (**b**) (001)_*γ*_//(021)_*α*_ interface with its binding energy = 5.475 eV/atom. The scale of the electron density ranges from −0.03 electron/Å^3^(blue) to 0.03 electron/Å^3^(red).

To further look into the correlation between interfacial strength and EWF, we conducted first-principles analysis to look into variations in EWF across *α*/*γ* interfaces. Since we are interested in how the interfacial bonding strength is related to the work function rather than investigating specific interfaces, two interfaces consisting of low-index planes, (001)γ //(001)α and (001)γ //(021)α, were used for the analysis. The low-index surfaces, when joined, have relatively simple structures and low interfacial mismatches and stresses, which facilitate the analyses. Other pairs could also be used for the analysis. The interfacial strength is evaluated based on Griffith energy to destroy the interface. Figure 4 illustrates variations in EWF and electron density across two *α*/*γ* interfaces. Curves in blue represent the electrostatic potentials and red curves are work functions obtained by averaging the potentials. The sub-figures under the curves show variations in the electron density long [010] direction. Regions in blue are deficient in electrons while those in red are rich in electrons. The grey balls represent Fe ions.

EWF is the energy required to move electrons at Fermi level from inside a metal to its surface without kinetic energy. We calculated the potentials (negative values) along [001] direction. Deep potential-wells correspond to large energies for electrons to be pulled out to the vacuum, while smaller energies are needed for electrons escape from lower potentials-wells. The average absolute value of potential along [001] represents the corresponding electron work function. In Fig. 4, average values of absolute potentials along [001] direction marked by red-lines are EWFs in our interface model. Thus, EWF gradient values, e.g., *dφ*/*dx* = 0.31 eV/Å, shown in Fig. 4(a) equals the difference in EWF (Δ*φ* = 4.84–3.70 eV) between the two sides of the interface divided by the length of the interfacial region (Δ*x* = 3.68 Å).

As shown in Fig. 4, the (001)_*γ*_//(001)_*α*_ interface having a smaller EWF gradient (*dφ*/*dx* = 0.31 *eV*/Å) exhibits a stronger interface with its binding energy equal to 7.758 eV, while the (001)_*γ*_//(021)_*α*_ interface with a larger work function gradient *dφ*/*dx* = 0.8 *eV/*Å is weaker with a binding energy of 5.475 eV. It should be noted that there is a drop of EWF at (001)_*γ*_//(021)_*α*_ interface, which results from interfacial mismatch, leading to loose structure (see Fig. 4(b)). A loose structure is generally weaker with a lower electron density, corresponding to a lower EWF. This can be seen from the following analysis. As shown in Fig. 4(b), the trend of variations in EWF is similar to that for the electron density. This happens because the work function (*φ*) is correlated to the electron density (*ρ*_*e*_) with a relationship^20^ as $\phi \propto {\rho }_{e}^{1/6}$. A higher electron density corresponds to a higher work function. The electron density and its value and variation at interface affect the interfacial bonding, which is related to the electron density gradient: $d\phi /dx\propto d/dx({\rho }_{e}^{1/6})\propto (1/6{\rho }_{e}^{5/6})d{\rho }_{e}/dx$. The drop in EWF at interface comes from lattice mismatch between two adjacent phases^38^, which results in interfacial strain or defects such as dislocations and vacancies. A loose interfacial structure has a lower electron density, which further increases the work function gradient as the denominator in the above equation is smaller. As a result, the local EWF gradient rather than average one should be used. For instance, in Fig. 4(b), *dφ*/*dx* = 0.8 *eV*/Å rather than *dφ*/*dx* = 0.26 *eV*/Å should be used for evaluating the interface. Or in other words, for a poor interface with a large lattice mismatch and resultant defects, the work function drops in the interfacial zone, which could be treated as a layer having a different configuration. In this case, the interfacial bonding strength should be dominated by the weakest region, corresponding to the steepest change in local work function i.e. the largest local work function gradient.

For a strong interface, strong interaction or “communication” between two sides of the interface results in a gradual change in EWF across the interface. If the interaction is weak, change in EWF across the interface should be steep. Steep variations in EWF are observed for incoherent interfaces and grain boundaries^27, 38^. The first-principle analysis supports the theoretical analysis, though further studies are needed in order to establish quantitative relationship between values from the experimental measurement and computational analysis.

**Figure 5:**
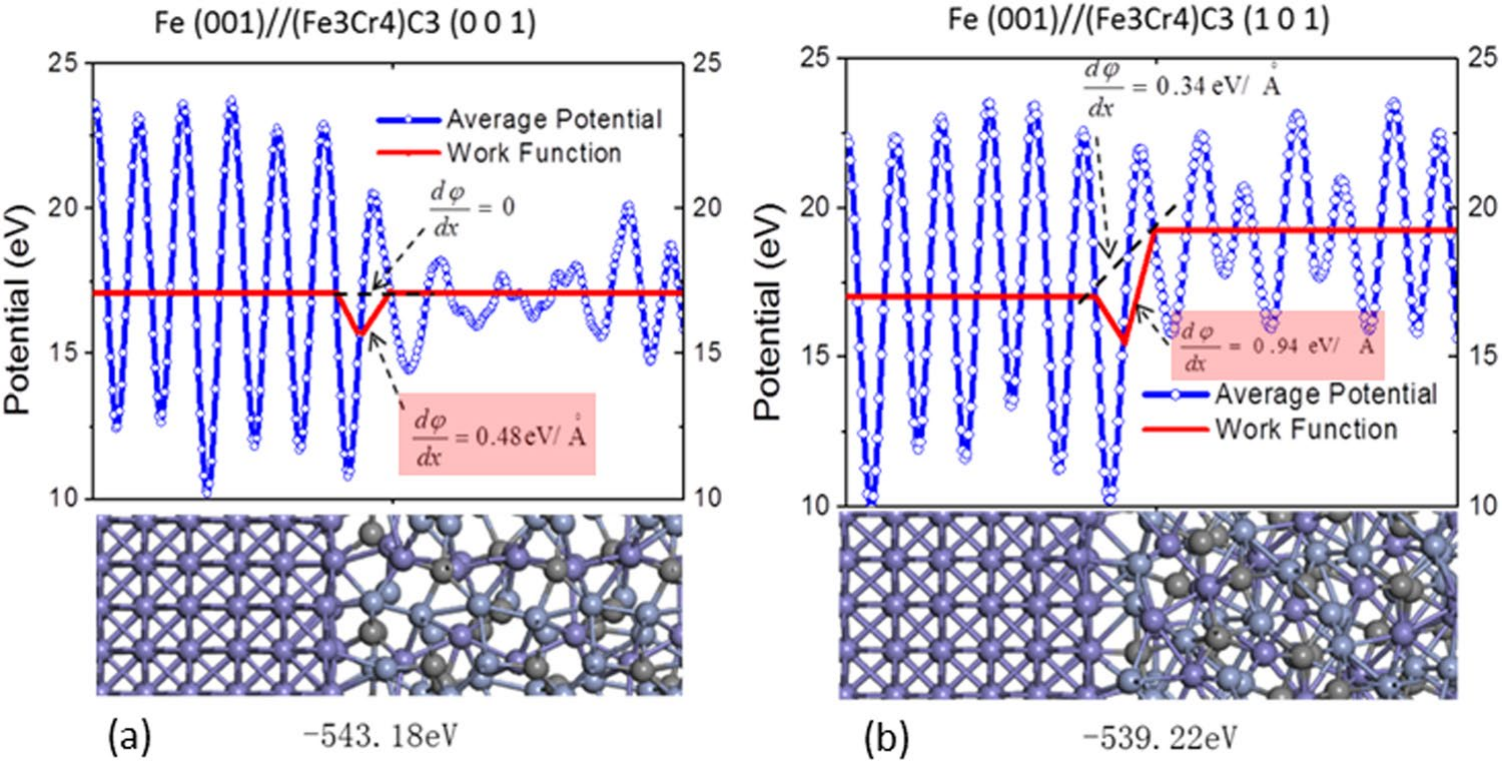
Potential and EWF in the interfacial region of (**a**) *Fe*(001)//(*Fe*_3_ *Cr*_4_)*C*_3_(101), and that of (**b**) *Fe*(001)//(*Fe*_3_ *Cr*_4_)*C*_3_(001). The lower portion of each figure shows the corresponding atomic configuration.

We also analyzed interfaces between (*Fe*_3_ *Cr*_4_)*C*_3_ and Fe with two orientation relationships: *Fe*(001)//(*Fe*_3_ *Cr*_4_)*C*_3_(101) and *Fe*(001)//(*Fe*_3_ *Cr*_4_)*C*_3_(001). Figure 5 shows interfacial structures, potentials and EWFs, and corresponding binding energies. Compared to the former, the latter is stronger with a smaller $d\phi /dx=0.48eV/\mathop{{\rm{A}}}\limits^{\circ }$ corresponding to a larger binding energy of 543.18 eV/cell.

**Figure 6:**
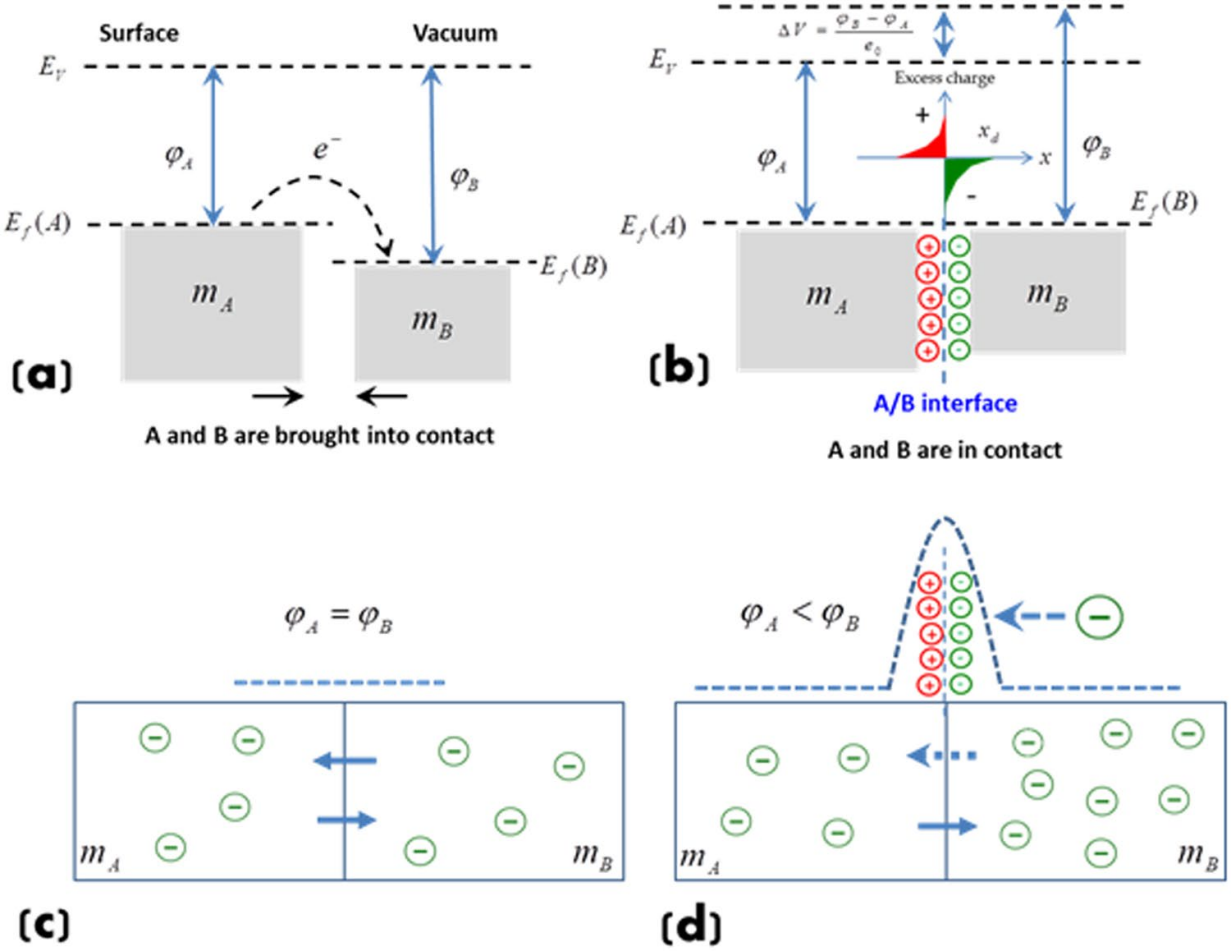
(**a**) Metals A and B are brought into contact, electrons migrate from A to B due to *φ*_*A*_ < *φ*_*B*_; (**b**) When A and B in contact, an interfacial dipole layer is established, driven by the potential difference, Δ*V*; (**c**) If A and B are identical, there is no a dipole layer and electrons can move freely across the interface; (**d**) If A and B are dissimilar, electrons tend to move from metal B with a higher electron density to metal A. On the other hand, the difference in EWF drives electrons to move from metal A to B. As a result, the formed dipole layer would act as a barrier to electron migration, thus negatively affecting the development of interfacial bonding.

In order to understand the correlation between interfacial bonding and EWF or the electronic origin of interfacial strength, Fig. 6 illustrates what may happen as two metallic phases are in contact. As shown in Fig. 6(a and b), when two different metals are brought into contact, their Fermi levels must become equal^39, 40^. Thus, electrons will move from the metal with a lower work function (*φ*_*A*_) to that having a high one (*φ*_*B*_), driven by a potential difference, Δ*V* = (*φ*_*B*_ − *φ*_*A*_)/*e*_0_, where *e*_0_ is the unit charge. As a result, a dipole layer will form at the A/B interface. If A and B are two pieces of identical metals, there is no a dipole layer and electrons can move freely across the interface, thus establishing metallic interaction or bonding, forming an integrated system as Fig. 6(c) shows. However, when two dissimilar metals are in contact, free electrons tend to locally redistribute, moving from the metal with a higher electron density to the one with a low electron density. However, on the other hand, the difference in EWF drives electrons to move from the metal having a low EWF to that having a high EWF. As a result, the dipole layer would act as a barrier to the electron migration, thus negatively affecting the development of interfacial bonding (Fig. 6(d)). Therefore, for a pair of dissimilar metals, the larger the difference in their EWF, the larger the barrier to the electron redistribution. Using the pair of identical metals with zero interfacial EWF (*dφ*/*dx*|_int_ = 0) as a reference, a larger *dφ*/*dx*|_int_ implies weaker “communication” between the two pieces of metals having a larger degree of dissimilarity. If there is no interaction, *dφ*/*dx*|_int_ = ∞. $\frac{d\varphi }{dx}{|}_{\mathrm{int}}\to \infty$ is an extreme case. Very dissimilar materials may still have strong interactions. For instance, carbides in cast irons show good bonding with the ferrous matrix, since the carbides have metallic bond components^41, 42^, leading to interactions with the metallic matrix. Van deer Waals interaction may also contribute to the interfacial bonding. For completely non-conductive materials, if there is no charge redistribution, interfacial bonding is hard to be established.

The interfacial work function gradient is determinable. As Fig. 6(b) illustrates, there are charge depletion zones with opposite signs at two sides of the interface. The depletion width, *x*_*d*_, is proportional to the square root of the difference in EWF between the metals^43, 44^ i.e. ${x}_{d}^{2} \sim {\rm{\Delta }}\phi ={\phi }_{B}-{\phi }_{A}$. Thus, one may represent the EWF gradient at the interface as ${d\phi /dx|}_{\mathrm{int}}\approx {\rm{\Delta }}\phi /2{x}_{d}={\rm{\Delta }}\phi /2\sqrt{{\rm{\Delta }}\phi }=\sqrt{\Delta \phi }/2$. Such a relationship indicates that when two metals having a larger difference in EWF, a larger interfacial EWF gradient is generated, corresponding to weaker interfacial bonding. If one of the materials is non-conductive, no charge transfer is possible, leading to an infinite EWF gradient without forming interfacial bonding. For an interface with large lattice mismatch, one may treat the interfacial region as a layer of different material with high disordering. The above discussion or theoretical consideration is still applicable.

It should be pointed out that the formation of the diploe layer causes local charge redistribution, which is related to local atomic displacement or local oscillations of interplanar distances^45, 46^ and thus generates interfacial strain. From the view-point of thermodynamics, if the two dissimilar materials have little interaction, no or little local charge redistribution would occur at the interface, corresponding to a large interfacial EWF gradient, *dφ*/*dx*|_int_ and a high interfacial energy (*γ*_*AB*_). If the dissimilar materials chemically interact but no sufficient local lattice relaxation occurs (corresponding to high *dφ*/*dx*|_int_), the large interfacial misfit strain helps increase the interfacial energy (*γ*_*AB*_). As a result, the work of adhesion, *W*_*AB*_ = *γ*_*A*_ + *γ*_*B*_ − *γ*_*AB*_ (*γ*_*A*_ and *γ*_*B*_ are surface energies of materials A and B), would be low, corresponding to weak interfacial bonding. If the interfacial structure can be relaxed with minimized interfacial strain towards equilibrium, stronger bonding with a lowered interfacial EWF gradient is expected. We would like to indicate that though the local charge redistribution is complicated, depending on the specific material pair and bond type^45–47^, the proposed mechanism for the interfacial bonding should be a general view related to electronic process for the development of interfacial bonding.

In conclusion, we demonstrate a novel method to diagnose local interphase boundary based on interfacial electron work function (EWF) and its gradient across the interface ($\frac{d\phi }{dx}{|}_{\mathrm{int}}$), which can be analyzed using a nano-Kelvin probe with atomic force microscope. It is shown that that a strong interface has its electron work function gradually changed across the interface, while a weaker one shows a steeper change in EWF across the interface. Besides, EWF may drop at a poor interface, e.g., an incoherent interface where interfacial defects lead to lowered electron density, since EWF is related the electron density. In this case, the drop of EWF caused by the interfacial defects enlarges the local $\frac{d\phi }{dx}{|}_{\mathrm{int}}$, thus further weakening the interface. Both experimental and theoretical studies confirm that the interfacial work function gradient is a measure of the interaction between two sides of the interface. The effectiveness of this method is demonstrated by analyzing sample metal-metal and metal-ceramic interfaces. Underlying mechanism is elucidated.
